# Time series analysis and short-term forecasting of monkeypox outbreak trends in the 10 major affected countries

**DOI:** 10.1186/s12879-023-08879-5

**Published:** 2024-01-02

**Authors:** Tahir Munir, Maaz Khan, Salman Arif Cheema, Fiza Khan, Ayesha Usmani, Mohsin Nazir

**Affiliations:** 1 Department of Anaesthesiology, Aga Khan University Hospital, Private Wing, Second Floor, Stadium Road, PO. Box 3500, Karachi, 74800 Pakistan; 2 Department of Applied Sciences, National Textile University, Faisalabad, 37610 Pakistan

**Keywords:** Time Series Modelling, Monkeypox, ARIMA, Forecasting, Viral Transmission

## Abstract

**Background:**

Considering the rapidly spreading monkeypox outbreak, WHO has declared a global health emergency. Still in the category of being endemic, the monkeypox disease shares numerous clinical characters with smallpox. This study focuses on determining the most effective combination of autoregressive integrated moving average model to encapsulate time dependent flow behaviour of the virus with short run prediction.

**Methods:**

This study includes the data of confirmed reported cases and cumulative cases from eight most burdened countries across the globe, over the span of May 18, 2022, to December 31, 2022. The data was assembled from the website of *Our World in Data* and it involves countries such as United States, Brazil, Spain, France, Colombia, Mexico, Peru, United Kingdom, Germany and Canada. The job of modelling and short-term forecasting is facilitated by the employment of autoregressive integrated moving average. The legitimacy of the estimated models is argued by offering numerous model performance indices such as, root mean square error, mean absolute error and mean absolute prediction error.

**Results:**

The best fit models were deduced for each country by using the data of confirmed reported cases of monkeypox infections. Based on diverse set of performance evaluation criteria, the best fit models were then employed to provide forecasting of next twenty days. Our results indicate that the USA is expected to be the hardest-hit country, with an average of 58 cases per day with 95% confidence interval of (00—400). The second most burdened country remained Brazil with expected average cases of 23 (00—130). The outlook is not much better for Spain and France, with average forecasts of 52 (00—241) and 24 (00—121), respectively.

**Conclusion:**

This research provides profile of ten most severely hit countries by monkeypox transmission around the world and thus assists in epidemiological management. The prediction trends indicate that the confirmed cases in the USA may exceed than other contemporaries. Based on the findings of this study, it remains plausible to recommend that more robust health surveillance strategy is required to control the transmission flow of the virus especially in USA.

**Supplementary Information:**

The online version contains supplementary material available at 10.1186/s12879-023-08879-5.

## Introduction

Monkeypox (Mpox) is a relatively rare zoonotic disease,caused by the Mpox virus, a virus closely related to the variola virus (responsible for the small-pox disease) by belonging to the same genus of Orthopoxviruses [1]. As suggested by the name, the monkeypox virus was first discovered during an outbreak amongst monkeys at a Danish laboratory in 1958 [2], but it was not until 1970 when the first human, a 9-month-old baby, was diagnosed in the Democratic Republic of Congo (formerly Zaire) [3], and since then been referred to as human Mpox virus.

Mpox has been endemic to West and Central Africa, with the most affected country being the Democratic Republic of Congo (DRC), where regular outbreaks have been the norm for the past five decades [1, 4, 5]. More recently the WHO reported 4,594 new suspected cases between January and September 2020, in the DRC alone, suggesting a steady rise in incidence [6]. This was followed by cases being reported in other parts of the world with around 3413 Mpox virus infections being reported across 50 countries. This led to the WHO declaring Mpox as an “evolving threat of moderate public health concern” in June 2022 [7, 8].

Till the 1980s transmission to humans originated from contact with wildlife reservoirs [9, 10]. More recently, most cases outside of Africa were due to animal-to-human transmission, imported from endemic countries, or associated with imported pets [1, 11, 12]. Only in the 1990s when the number of secondary cases by contact with an infected person began to increase was Mpox considered an important worldwide health concern [10]. The transmission of the virus occurs mostly through large respiratory droplets, close or direct contact with skin lesions, and possibly through contaminated fomites [7, 13]. Vertical transmission and fatal deaths have also been described [14]. The current spread has been shown to disproportionately affect men who are gay or bisexual and other men who have sex with men (GBMSM), which may suggest amplification of transmission through sexual networks [15]. According to the UK Health Security Agency, of the 152 male confirmed reported cases 151 were identified as GBMSM [16]. At present, it is still not clear whether Mpox can be transmitted through semen or vaginal fluid.

Although not as severe, Mpox disease shares many clinical characteristics with the smallpox disease such as an initial febrile prodrome lasting between 1 to 4 days with generalized headaches and fatigue. The initial prodromal period is followed by (or concomitant with) the development of a maculopapular rash, that often first appears on the face and then appears in a centrifugal distribution on the body [17, 18]. These lesions may occur in the oral cavity causing difficulty swallowing [17]. The disease also characteristically results in maxillary, cervical, or inguinal lymphadenopathy which is unique when compared to smallpox and suggests a more effective immune recognition and response (a hypothesis that requires further study) [17, 19]. Smallpox (*Variola major*variant) had a case fatality rate of 30%, fortunately the symptoms from Mpox disease are much less severe and self-limiting lasting with symptoms usually resolving within 2 to 4 weeks. Severe cases usually only appear in children and immunocompromised. Complications are rare but include pneumonitis, encephalitis, keratitis, and secondary bacterial infections [7].

The clinical differential diagnosis includes other rash illnesses, such as chickenpox, measles, bacterial skin infections, scabies, syphilis, and medication-associated allergies. All suspected cases of Mpox disease should be reported immediately to a local health department for proper infection control and contact tracing but given the current rarity of the disease and wide range of clinical differential diagnoses, reaching a diagnosis of Mpox poses a challenge for physicians.

Combined with clinical and epidemiological information diagnostic assays are the most powerful and important components for the identification of Orthopoxviruses as recommended by the WHO [19, 20]. McCollum et al [17] discussed the pros and cons of multiple diagnostic assays, the most reliable of which is real-time polymerase chain reaction (PCR). These assays are highly sensitive and efficiently detect viral DNA but require high-quality laboratories that either use in rural and low-resource regions [17, 20].

The COVID-19 outbreak in 2019 has changed the way we view zoonotic infections but experts have been warning the public about the threat of zoonotic infections as far back as 2003 during the 2003 SARS outbreak [21]. The fear of another pandemic has led to taking measures to control the disease early on. Statistical analyses play an important role in the prediction of disease spread and can help prepare and control outbreaks through planning and policies. Since its inception as a field of study more than a century ago, infectious disease epidemiology has placed a high priority on the statistical representation and analysis of infectious diseases [22, 23]. In recent years, in particular, for newly emerging disease outbreaks, forecasting modelling is in great demand and significantly contributes to emerging disease outbreaks and public health [8, 24, 25]. The goals of modelling include identifying epidemiological characteristics to comprehend infectious diseases, forecasting disease trends, assessing control measures to guide decision-making, and investigating uncertainty. To study the spread of infectious diseases, numerous models have been developed, examined, and used [8, 26–31], a few cited therein.

The time series modelling has long and rich history in epidemiological pursuits. For example [32], studied the dynamics of influenza epidemics in space and time where disease counts were considered to follow multivariate autoregressive process. Further [28], proposed a dynamic model based on SIR-type differential equation to enumerate impact of early health interventions in context of COVID-19 pandemic. Furthermore, SIR model was used by [26] to predict that H1N1 (the swine flu) would pose a serious threat to Israel's public health. Moreover [29], used the Poisson-lognormal, Poisson-generalized Gamma, and Poisson-Weibull distributions to enumerate the spread proportion of COVID-19 in Hong Kong, India, and Rwanda. Also, using a zero-truncated negative binomial model [33], conducted a study to infer the super spreading potential COVID-19 flow around the globe. Additionally [34], proposed a new zero-state coupled Markov switching negative binomial model in which the disease alternates between periods of presence and absence in each area using a series of partially hidden nonhomogeneous Markov chains coupled between nearby locations. The distribution of COVID-19 confirmed cases in China was examined by [35] With respect to the dynamics of Power law.

Mpox cases, on the same lines, are time-series data with some dynamic fluctuation trend in the various circumstances with epidemic prevention and control, making it appropriate to create a time-series model for prediction. Predicting the daily new cases and total confirmed cases of Mpox for all the most affected countries is therefore extremely important from a practical standpoint.

However, as time-series data, Mpox cases have some dynamic fluctuation trends in the various situation with epidemic prevention and control, which is suitable for establishing a time-series model for prediction. The Automatic Regressive Integrated Moving Average (ARIMA) model, which has a simple structure and immediate applicability, is one of the most popular time series models. The capability of ARIMA model in extracting the trends in the data by considering moving averages and then obtain of the stationarity of the series by differentiating, is well documented in research literature [36]. The ARIMA model has been widely used to predict and estimate the prevalence of common diseases, including COVID-19 [37, 38], typhoid fever [39], tuberculosis [22], and influenza [23, 40, 41]. ARIMA methods are capable of correlating regulation with short-term changing trends in time series despite their lack of reliance on mathematics and statistics. Therefore, the model is more suitable for predicting short-term epidemic diseases.

Therefore, it is of great practical significance to predict the daily new cases and cumulative confirmed cases of Mpox for all the most affected countries. This study develops best-fitted ARIMA models to predict daily new cases and cumulative confirmed cases of monkeypox in Spain, the United States, Germany, the United Kingdom, France, the Netherlands, Colombia, Mexico, Brazil, and Canada over the next 20 days and evaluates the model's prediction accuracy to provide a further reference for the prediction and early warning of infectious diseases. These models may also be used to predict the more days by incorporating the more days of data.

## Material and methods

### Data collection

**Fig. 1:**
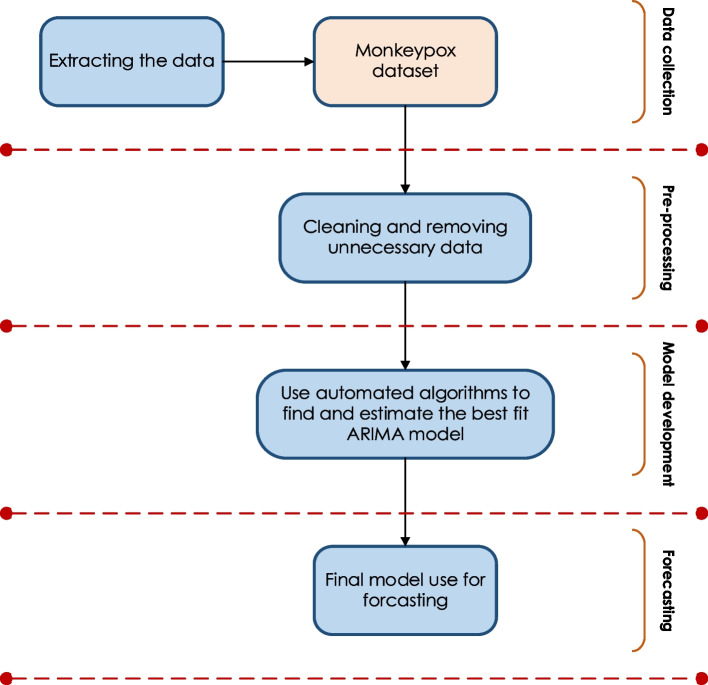
The proposed methodology of the Monkeypox forecasting flow

**Fig. 2:**
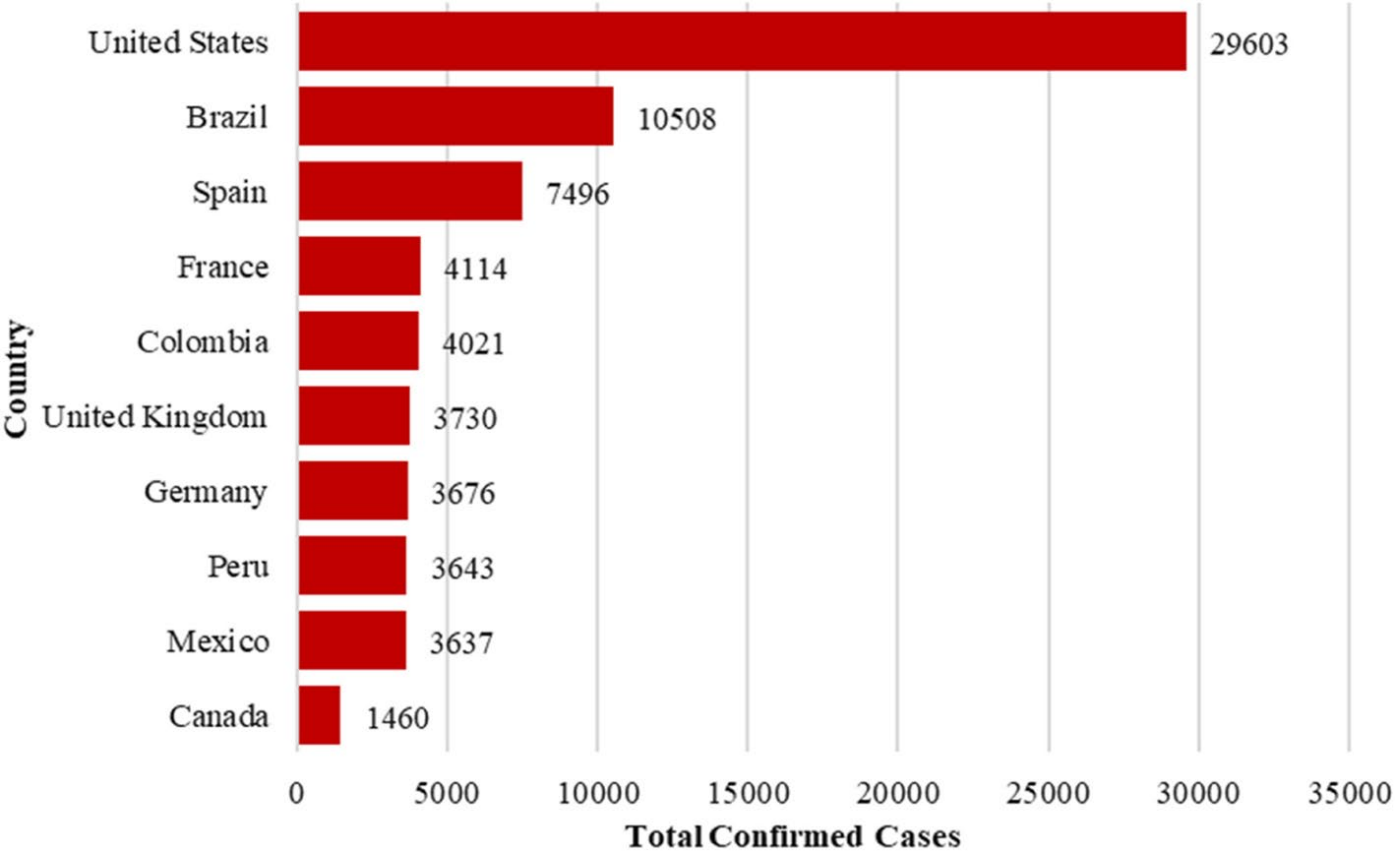
Monkeypox cases in the eight most affected countries

**Fig. 3:**
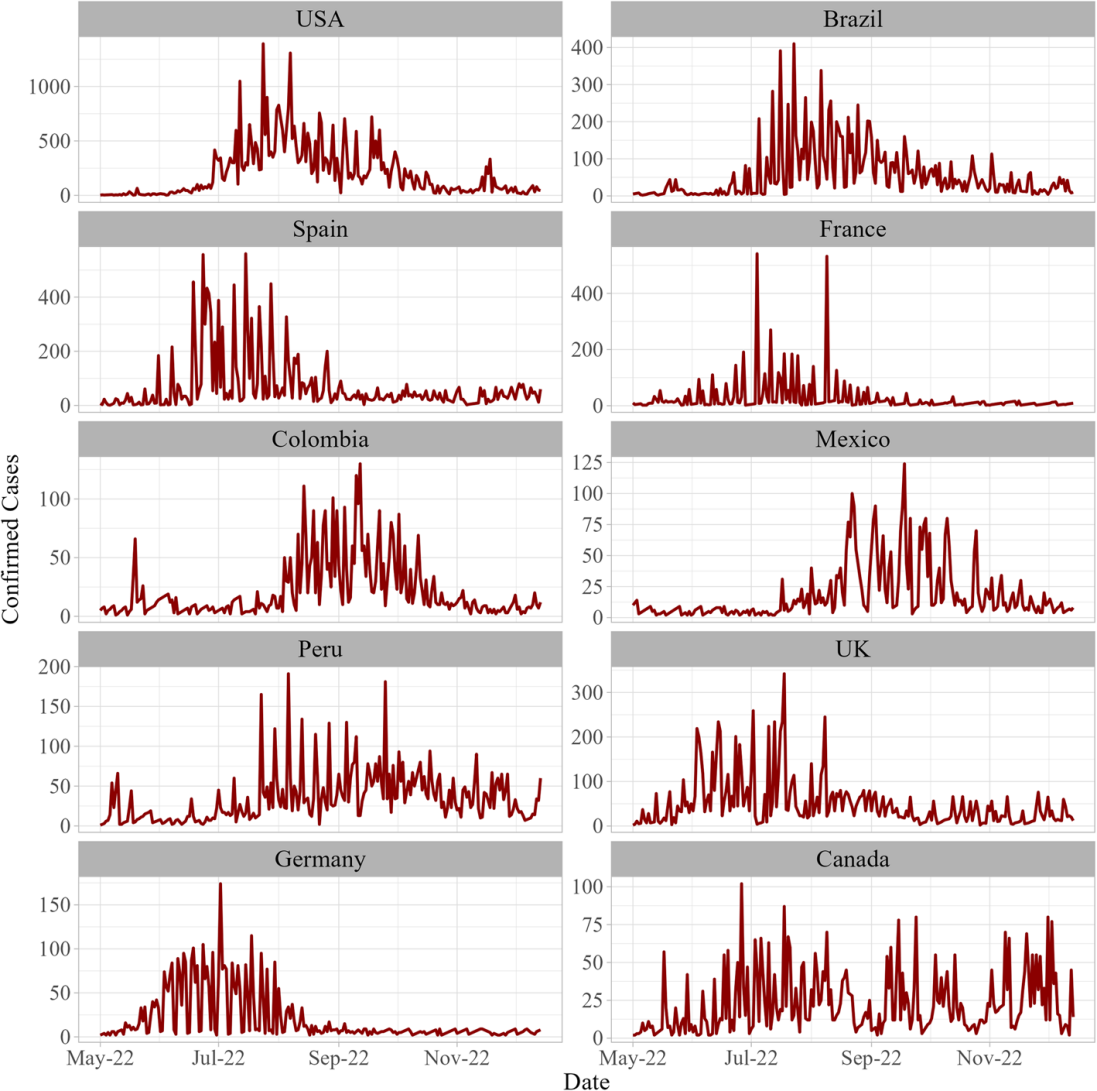
Comparison of daily new confirmed Monkeypox cases in most eight affected countries from May 16, 2022, to July 25, 2022

**Fig. 4:**
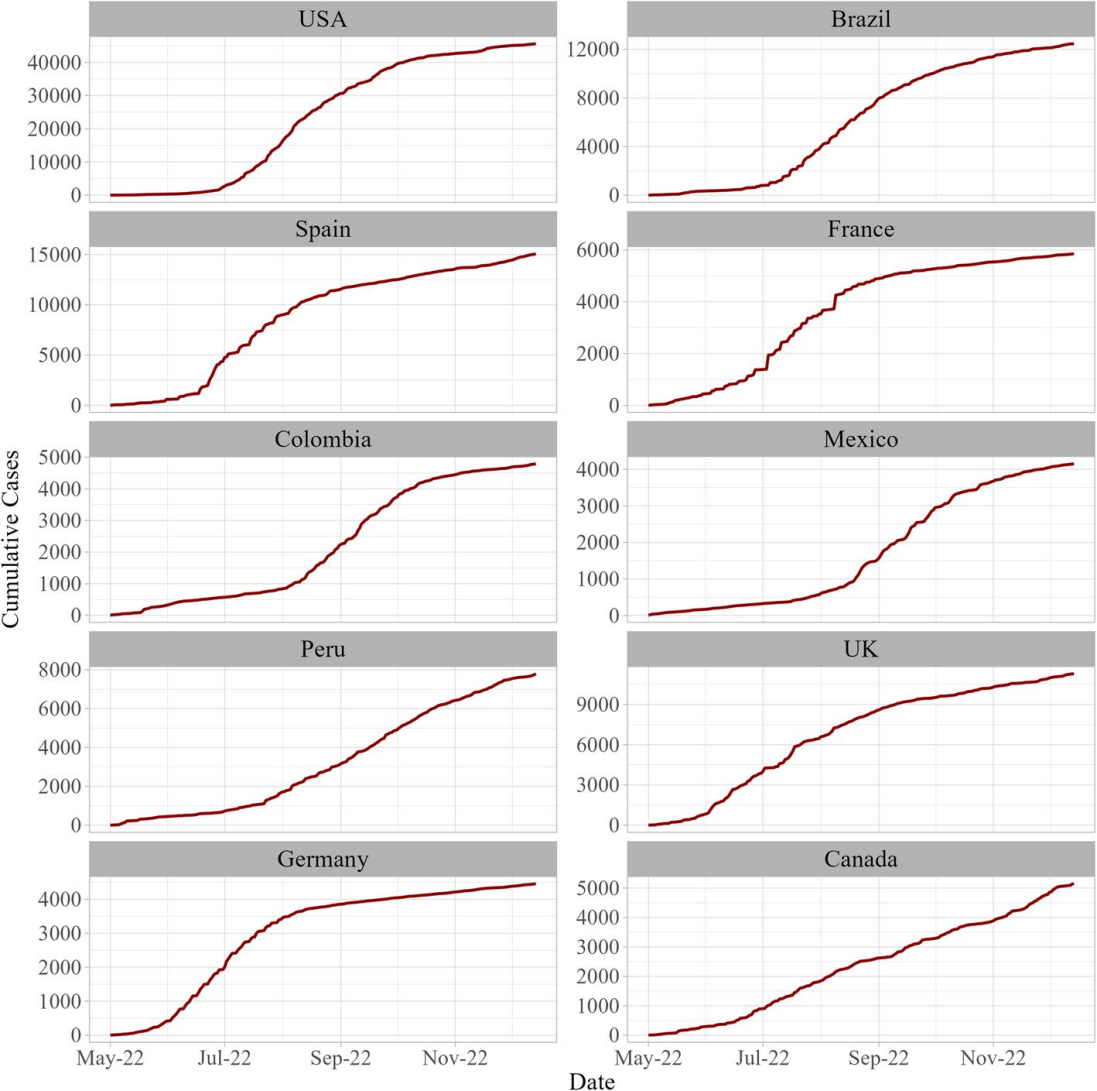
Daily variation cumulative confirmed Monkeypox cases in most eight affected countries from May 16, 2022, to July 25, 2022

The data for this study were collected from the official Our World in Data website (https://ourworldindata.org/). Data consisted of daily confirmed cases and cumulative cases of Mpox disease from the eight most affected countries, namely Spain, United States, Germany, United Kingdom, France, Netherlands, Brazil, and Canada, from May 18, 2022, to December 31, 2022. We dropped the initial ten days due to zero-inflated observations in all considered countries to develop stable and effective forecasting time series models. The data extraction methodology is also discussed in Fig. 1. The considered dataset (May 16, 2022, to December 31, 2022) was then used to forecast new confirmed and cumulative disease cases across the countries over the next 20 days (January 01, 2023, to January 20, 2023). Supplementary Table S1 and Figs. 2, 3 and 4 provide a statistical descriptive analysis of this raw data.

### ARIMA model

One of the well-known and widely applied statistical techniques for time-series forecasting is the Auto Regressive Integrated Moving Average (ARIMA) model. The standard dependencies that are specific to time series data are captured by this class of statistical algorithms. The Box—Jenkin model, first presented by Box and Jenkins in the 1970s [42], is a type of algorithm for the analysis and forecasting of time series data. This model, which is used to estimate and extrapolate the state of something at some point in the future by analysing the pattern of historical data and making future predictions based on that pattern and historical data from the past and the present, is applied to non-stationary time series after smoothing the data [43]. The model can be regarded as suitable for forecasting because the daily new cases and total number of confirmed cases of monkeypox are random series with nonlinear or seasonal character. ARIMA simulates and predicts how series of data will be in the future. The following actions are part of the ARIMA model [44]:

- Step 1: Determining the functional form of the model.
- Step 2: The estimation of the model's parameters.
- Step 3: Verify the model validation hypotheses.
- Step 4: Modelling predictions is step four.

The ARIMA (p, d, q) model in general has the structure as of Eq. (1).

1 $${y}_{t}={\phi }_{1}{{\text{y}}}_{t-1}+{\phi }_{2}{{\text{y}}}_{t-2}{+\dots +\phi }_{p}{{\text{y}}}_{t-p}{-\uptheta }_{1}{\upvarepsilon }_{t-1}-{\uptheta }_{2}{\upvarepsilon }_{t-2}\dots -{\uptheta }_{q}{\upvarepsilon }_{t-q}.$$

The parameters of the model are ${\phi }_{{\text{a}}}({\text{a}}=\mathrm{1,2},\dots ,p)$ and ${\uptheta }_{b}(b =\mathrm{0,1},2,\dots ,q)$ in Eq. (1). ${{\text{y}}}_{{\text{t}}}$ and ${\upvarepsilon }_{{\text{t}}}$ represents the starting value and random error at time step ${\text{t}}$, respectively. With a mean and standard deviation of zero, the arbitrary error represented by ${\upvarepsilon }_{{\text{t}}}$ represents the ${\upsigma }^{2}$. When q is set to 0, Eq. (1) functions as an A.R. model with order p, and when $p$ is set to 0, it transforms into an M.A. model with order $q$. Therefore, ($p,q$) are both significant parameters in establishing the ARIMA model.

### Analytical tools and model evaluation

#### ACF and PACF test

The autocorrelation value for any sequence with lag values is given to us by the ACF, which is a complete autocorrelation function. It briefly describes how closely that sequence's present value and its past value are correlated. PACF (Partial Autocorrelation Function) and determines the correlation between the residuals and the subsequent lag value rather than lags like ACF with the current. The linear relationship between the observations at time $t$ and the observations at time $t - n$ is displayed by an ACF. For a specific time series $X$, the ACF and PACF are defined as:

2 $$\left.\begin{array}{c}ACF\left({X}_{t},{X}_{t-n}\right)=\frac{Covariance\left({X}_{t},{X}_{t-n}\right)}{Variance\left({X}_{t}\right)}\\ PACF\left({X}_{t},{X}_{t-2}\right)=\frac{Covariance({X}_{t, }{X}_{t-2}/{X}_{t-1})}{\sqrt{Variance({X}_{t}/{X}_{t-1})} \sqrt{Variance({X}_{t-2}/{X}_{t-1})}}\end{array}\right\},$$

where $n$ is the lag (or difference between ${X}_{t}$ and ${X}_{t-n}$) in the ACF plot and $n=2$ in the PACF plot between the observed values ${X}_{t}$ and ${X}_{t-n}$.

#### Performance indices

To measure the effectiveness of a model fitting and forecasting, following are the indices: RMSE (root mean square error), MAE (mean absolute error), MAPE (mean absolute prediction error) and ME (mean error), were applied. They were used to assess how well the developed models predicted the future. A better data fit is indicated by lower values aforementioned indices. These criteria are expressed, respectively [43], in Eqs. (6 and 7). AIC (Akaike Information Criterion), and BIC (Bayesian Information Criterion) are information criterion classes used to assess the goodness of fit of a statistical model. It is based on the concept of entropy and can weigh the estimated model's complexity against its goodness of fit to the data. This data is used to evaluate the model's parameters and how well the model performed. To avoid excessive model complexity caused by excessive model accuracy in this study. As a result, the function returns the lower value.

3 $$RMSE=\sqrt{\frac{SSE}{n}} =\sqrt{\frac{\sum_{i=1}^{n}{({Y}_{i}-{\overline{Y} }_{i})}^{2}}{n}}$$

4 $$MAE=\frac{1}{n}\sum\nolimits_{i=1}^{n}|{Y}_{i}-{\overline{Y} }_{i}|$$

5 $$MAPE=\frac{100}n\sum\nolimits_{i=1}^n\left|\frac{\left(Y_i-{\overline Y}_i\right)}{Y_i}\right|$$

6 $$AIC=2n-2logL\left(\widehat{\theta }\right)$$

7 $$BIC=nlogN-2logL\left(\widehat{\theta }\right)$$

In Eqs. (3, 4 and 5), where ${Y}_{i}$ is the actual expected output, ${\overline{Y} }_{i}$ is the model’s prediction, $i = 1\dots n$ and $n$ is the number of observations. In Eqs. (6 and 7), $logL\left(\widehat{\theta }\right)$ is the likelihood function, N is the number of observations, and $n$ is the number of model parameters.

#### Data analysis

Both new confirmed cases and cumulative cases of monkeypox exhibit time series properties. For the following 20 days, daily new and cumulative cases were predicted using best fitted ARIMA models. R software with forecast package was used for forecasting. The generation of ARIMA model parameters generally involves the use of statistical methods like performing a difference to remove non-stationarity and plotting ACF and PACF graphs. Based on lower AIC and BIC, this package intuitively selects the optimal set of parameters ($$p,d,q)$$ for better forecasting.

One of the highly admired classes of statistical tools to capture the time-dependent delicacies prevalent in the data to assist the time-series forecasting is the ARIMA model [45]. In its simplest form, the ARIMA models facilitate the extrapolation of the future by simulating the patterns existent in the current state of matter while catering the time-related characters of the available information ^32,42.^ Without losing the generality, the order of an ARIMA model is commonly denoted as ARIMA (*p,d,q*). Here, *p* represents the degree of dependency of the current state on its lag values, and $q$ denotes the order of the moving average process highlighting the access of past forecast error. Lastly, $d$ shows the order of non-seasonal differences required to attain stationarity while integrating both auto-regressive and moving average parts. Formally the ARIMA model is written as;

8 $${\phi \left(B\right){\left(1-B\right)}^{d}y}_{t}=\theta \left(B\right){\upvarepsilon }_{t}.$$

Here, $B$ is back shift operator with $\phi \left(B\right)$ being auto-regressive operator such as, $\phi \left(B\right)=1-{\sum }_{j=1}^{p}{\phi }_{j}{B}^{j}$. Further, $\theta \left(B\right)$ represents the moving average operator where, $\theta \left(B\right)=1+{\sum }_{i=1}^{q}{\theta }_{i}{B}^{i}$ and $d$ indicates the non-seasonal differences to gain stationarity. The diversity and flexibility of ARIMA models are noteworthy as it enables the analyst to not only extrapolate integrated formations but also the moving averages and auto-regressive complexities alone as well as simultaneously. Due to these delicacies, the use of the ARIMA model is anticipated to be more elaborate in model and forecasting the trends of monkeypox transmission.

### Analytical tools and model evaluation

#### Autocorrelation function (ACF) and partial autocorrelation function (PACF)

The lack of independencies and so the linear predictability of the current state based on lagged values is assessed by considering ACF. A more elaborative account of dependencies is offered while holding all other mediators constant by the launch of PACF.

#### Performance indices

The performance evaluation of the most prominent models is investigated on multiple fronts by considering numerous measures. The predictive capability of the models is assessed by considering the relevant indices such as RMSE, MAE, MAPE and ME. Whereas the loss of information concerning parametric estimation is enumerated by using entropy-based statistics such as AIC, BIC, and Corrected AIC (AICc) [42, 43].

## Results and discussions

### Exploratory analysis

The dynamic characteristics of the transmission of Mpox in eight countries are investigated by the launch of the well-celebrated general scheme of the ARIMA model. The data regarding the daily reported new cases and cumulative frequencies ranging from May 18, 2022, to December 31, 2022, were assembled from the official website of Our World in Data. The exploratory analysis reveals that the greatest number of confirmed cases during the above-mentioned period was reported in United States which is 29,603 (Fig. 2).

It was then followed by the Brazil (10,508), Spain (7496), France (4114) and Colombia (4021). Where, United Kingdom, Germany, Peru and Mexico all showing more than 3000 confirmed cases of the outbreak. Lastly, Canada reveals confirmed cases of Mpox virus infection as 1460. The display of daily reported cases data convincingly exhibits the time-inflicted characters which are then aggregated in cumulative cases. The time series display daily cases reported in the selected countries showed that the reported numbers gained momentum after the second week of June, however, intensities vary (see Figs. 3 and 4).

These findings remain verifiable from the graph of cumulative frequencies indicating a rise in the degree of flow with varying degrees (Fig. 4). The numerical summaries of the data also pass various behavioural aspects of the time-dependent flow of the viral transmission (Supplementary Table S1).

On average United States seals its most affected situation concerning the outbreak with the highest mean value of 199 cases per day over eight months period. This is then followed by the Brazil, Spain, France, Germany, and the United Kingdom. Whereas Canada showed a minimal average value of almost 9 cases per day. Moreover, the highest number of newly reported cases in a single is also associated with United States as 1500 cases. This number is overwhelmingly distinctive from the following country of France and Spain with a maximum number of cases of 526 and 520 in one day. The least affected Canada projected the maximum number of reported cases associated with a single day as 106.

### Dynamic modelling

The dynamic parametric estimation and modelling of the time-dependent viral flow of the Mpox data related to eight selected countries forwarded different degrees of complexities. Both new confirmed cases and cumulative cases of Mpox exhibit time series properties. For the following 20 days, daily new and cumulative cases were predicted using best-fitted ARIMA models. RStudio (version 4.1.2; RStudio, Boston, MA, USA) with forecast package was used for forecasting.

**Fig. 5:**
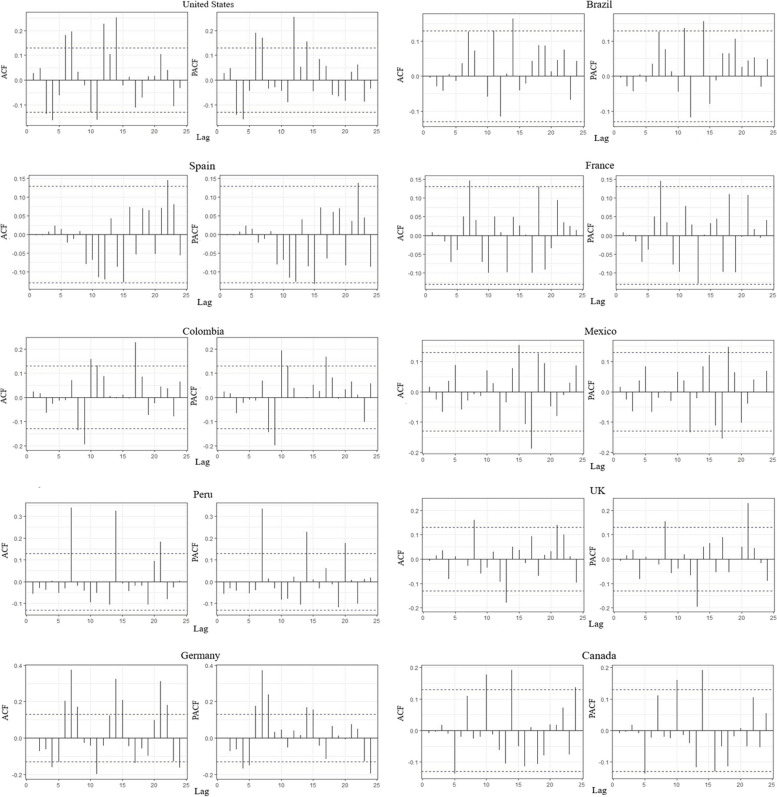
ACF and PACF of the residuals of the best fitted ARIMA models on the confirmed monkeypox cases

**Fig. 6:**
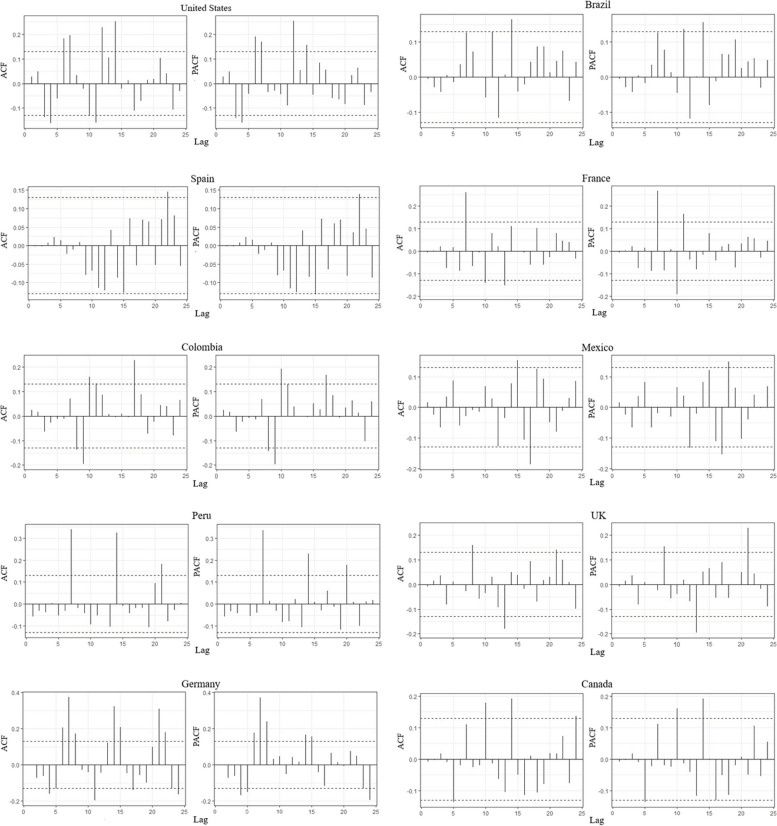
ACF and PACF of the residuals of the best-fitted ARIMA models on the cumulative monkeypox cases

The generation of ARIMA model parameters generally involves the use of statistical methods like performing a difference to remove non-stationarity and plotting ACF and PACF graphs (Figs. 5 and 6). Based on lower AIC, AICc, and BIC, this package intuitively selects the optimal set of parameters (*p,d,q*) for better forecasting 38. This function searches for a range of $p, q$ values, after fixing d by Kwiatkowski-Phillips-Schmidt-Shin (KPSS) test. It chooses the model having the lowest AIC score.

The daily reported data of Spain is best modelled with ARIMA (5,1,3) indicating the prevalence of auto-regressive factors along with moving average part and non-seasonal effects. Similarly, ARIMA (2,1,2), ARIMA (2,1,1) and ARIMA (1,1,2) models are estimated concerning Germany, Netherlands, and Canada. Further, the US, France, and Brazil showed a lack of auto-regressive part in the modelling of daily reported data with estimated models such as ARIMA (0,1,2), ARIMA (0,1,1) and ARIMA (0,1,1), respectively. The UK data, however, distinctively exhibited the existence of an auto-regressive part only with the estimated model of the order ARIMA (4,0,0). The display of ACF and PACF indicates the linear dependency structure after the launch of the most appropriate models (Figs. 5 and 6).

The agreeable behavioural display can be noticed concerning each country’s estimation of daily reported data. Moreover, the model fitting and appropriateness criteria are compiled in the Table 1. One may notice the legitimacy of the estimated models on various fronts while trading off the multifaceted complexities of the viral flow of Mpox. As long as, the time-series modelling of cumulative cases data is concerned a relatively lesser extent of complexity is observed. This character can be attributed to the availability of more synchronized information. The ARIMA (2,2,2), ARIMA (2,2,1), ARIMA (1,2,1) and ARIMA (1,2,2) are estimated concerning Germany, Netherlands, Brazil, and Canada. Whereas ARIMA (0,2,3), ARIMA (0,2,2), ARIMA (0,1,1) and ARIMA (0,2,1) models gained prominence for Spain, US, UK, and France. The performance capacities of the argued models are substantiated while displaying the correlation formations through ACF and PACF (Figs. 5 and 6) along with relevant numerical summaries regarding the predictive power (Table 2).

**Table 1:** Estimation of optimal parameters for the best fitted ARIMA model Parameters and AICs of the ARIMA models for 8 countries

Country	Confirmed Cases			Cumulative Cases		
	**Model Structure**	**AICc**	**BIC**	**Model Structure**	**AICc**	**BIC**
United States	(0,1,1)	2956.78	2963.58	(0,2,1)	2944.78	2951.57
Brazil	(3,1,1)	2459.14	2476.00	(3,2,1)	2449.36	2466.19
Spain	(5,1,5)	2666.81	2706.45	(5,2,5)	2653.98	2690.37
France	(5,1,5)	2475.54	2511.99	(3,2,4)	2475.18	2501.88
Colombia	(2,1,3)	1941.21	1961.38	(2,2,3)	1933.68	1953.82
Mexico	(4,1,5)	1874.28	1907.52	(4,2,5)	1867.08	1900.27
Peru	(1,1,2)	2154.99	2168.51	(1,2,2)	2146.31	2159.81
United Kingdom	(1,1,1)	2408.85	2419.02	(1,2,1)	2399.16	2409.31
Germany	(2,1,1)	2042.54	2056.06	(2,2,1)	2034.56	2048.06
Canada	(2,1,1)	1993.57	2007.09	(2,2,1)	1985.74	1999.24

**Table 2:** Accuracy evaluation metrics of ARIMA models for forecasting Monkeypox cases

**Cases**	**Country**	**Model fitting**			
		**ME**	**RMSE**	**MAE**	**MAPE**
Confirmed	United States	1.0837	160.8289	93.6989	0.8021
	Brazil	0.6142	52.8620	32.3485	1.1673
	Spain	-0.0148	80.7937	43.7184	1.5725
	France	-0.0025	53.1115	23.8431	2.4846
	Colombia	0.0969	16.7897	11.1099	0.8945
	Mexico	-0.0376	14.2295	9.2355	0.8129
	Peru	0.8109	27.1984	17.0360	0.9097
	United Kingdom	0.5683	47.8629	30.2068	1.1890
	Germany	0.0949	21.2443	12.2563	1.3640
	Canada	1.2181	19.0702	13.9751	1.1023
Cumulative	United States	1.0546	160.8288	93.6859	1.7463
	Brazil	0.6121	52.8620	32.3425	1.8743
	Spain	0.7233	80.7973	43.5809	2.2172
	France	0.0659	55.0058	24.8170	1.8706
	Colombia	0.0835	16.7879	11.1024	1.3917
	Mexico	-0.0418	14.2278	9.2216	1.0140
	Peru	0.7166	27.1835	17.0301	1.6954
	United Kingdom	0.4919	47.8497	30.2065	1.7379
	Germany	0.0792	21.2429	12.2499	1.4778
	Canada	1.2083	19.0657	13.9674	1.5947

### Predictions

**Fig. 7:**
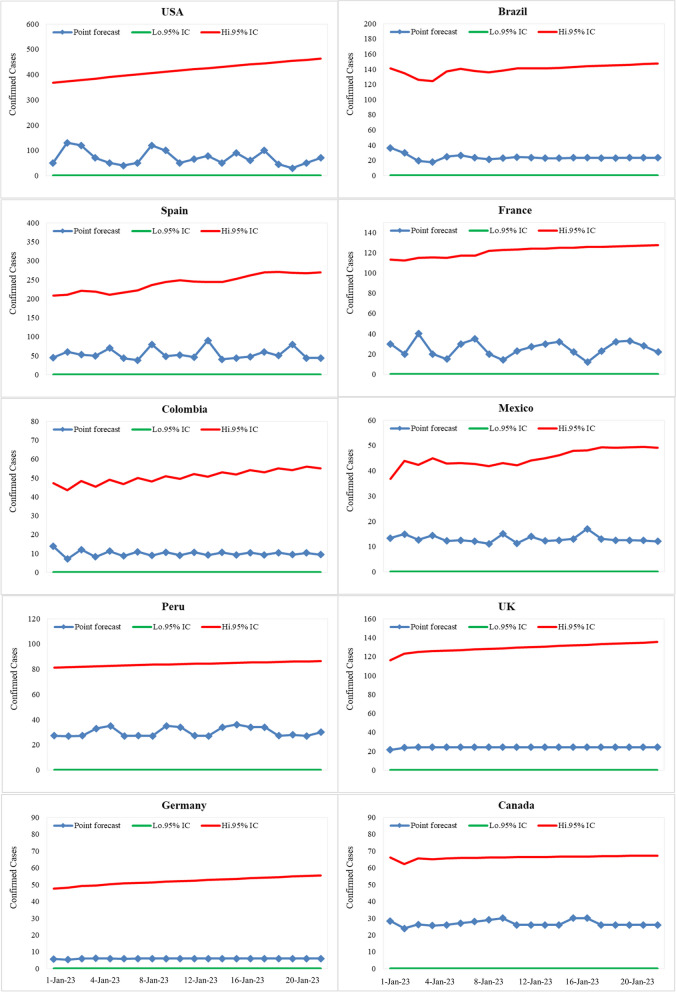
The prediction of the best fitted ARIMA model (95% confidence interval) for the daily new cases (for the next 20 days) from July 26, 2022, to August 13, 2022

**Fig. 8:**
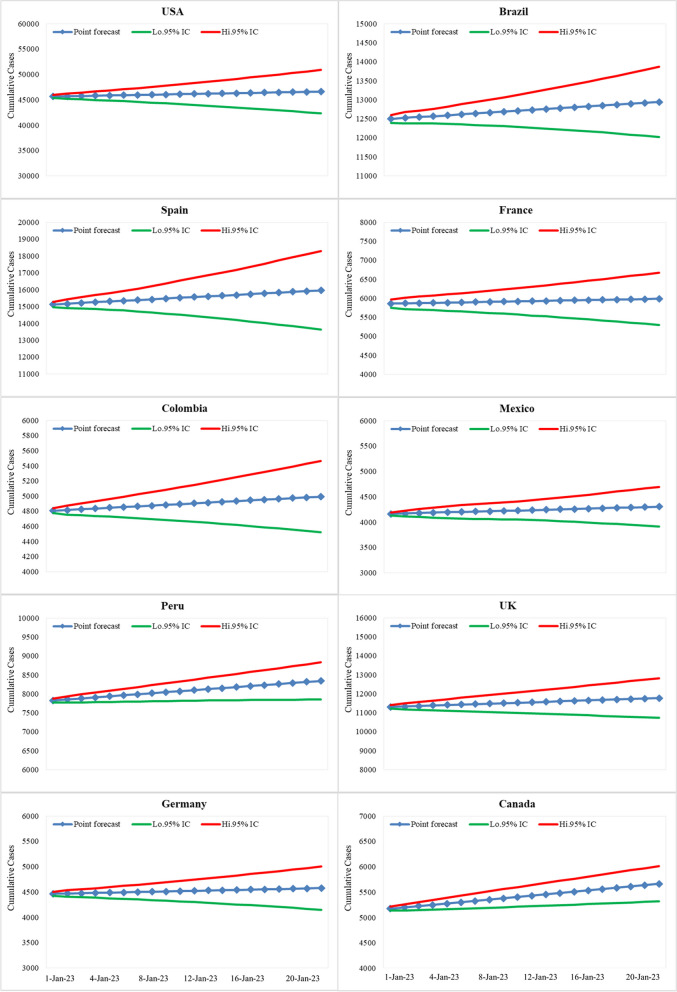
The prediction of the best-fitted ARIMA model (95% confidence interval) for the daily cumulative cases (for the next 20 days) from July 26, 2022, to August 13, 2022

For each country, the best fit ARIMA model was used to forecast the spread of Mpox in ten countries: United States, Brazil, Spain, France, Colombia, Mexico, Peru, United Kingdom, Germany and Canada. The data used in the models spanned from May 18, 2022, to December 31, 2022, with predictions made for the next 20 days till January 10, 2023, with 95% confidence intervals. The graphical display offered in Figs. 7 and 8 presents the predictive behaviour for daily reported data.

The results of the predictions showed a range of expected cases per day, with the United States estimated to have the highest number of cases at an average of 62 per day and the Germany expected to have the lowest at 7 per day. Meanwhile, countries like Peru, Brazil, France, the United Kingdom, and Canada, are forecasted to have averages of 32, 23, 24, 24, and 26 cases per day, respectively. Colombia, and Mexico are expected to have numbers of cases at 12, and 12 cases per day, respectively. The results of the predictive analysis show a need for each country to prepare for their respective levels of risk to control the spread of Mpox.

## Conclusions

In this study, the 10 most affected countries— United States, Brazil, Spain, France, Colombia, Mexico, Peru, United Kingdom, Germany and Canada—were examined about the current and short-term predicted possible daily confirmed and cumulative cases of the Mpox epidemic. The persistent trend and scope of the epidemic were estimated using ARIMA models. It has been revealed that, among other countries, the United States will most likely be affected by Mpox in the future, prompting people to be more vigilant of this virus.

The current work can help respected governments develop emergency plans and allocate medical resources. Whereas in this study, authors used data from almost three months to forecast the next twenty-day scenario. If the data set is sizable, it can also accurately predict long periods.

### Supplementary Information

**Additional file 1:** **Supplementary Table S1.** Descriptive statistics on the cases of Monkeypox in the 10 most affected countries.

## Data Availability

The data analyzed in this study were publicly available and retrieved from the official Our World in Data website at (https://ourworldindata.org).
