# Supplementary material for: Time series analysis and short-term forecasting of monkeypox outbreak trends in the 10 major affected countries
Source: BMC Infect Dis. 2024 Jan 2;24:16. doi: 10.1186/s12879-023-08879-5 (PMC10762824; doi:10.1186/s12879-023-08879-5)
Supplement: Supplementary file 1 — Additional file 1: Supplementary Table S1. Descriptive statistics on the cases of Monkeypox in the 10 most affected countries. [file 12879_2023_8879_MOESM1_ESM.docx]

**Supplementary Table S1:** Descriptive statistics on the cases of Monkeypox in the 10 most affected countries

| Cases | Country | Mean | SE | St. Dev | Minimum | Maximum | Skewness | Kurtosis |
| --- | --- | --- | --- | --- | --- | --- | --- | --- |
| Confirmed | United States | 199.98 | 15.69 | 236.89 | 0 | 1392 | 1.94 | 4.69 |
|  | Brazil | 54.66 | 4.62 | 69.73 | 1 | 410 | 2.33 | 6.30 |
|  | Spain | 66.11 | 6.62 | 100.01 | 1 | 560 | 2.90 | 8.46 |
|  | France | 25.67 | 4.02 | 60.73 | 0 | 541 | 5.96 | 43.17 |
|  | Colombia | 21.02 | 1.64 | 24.78 | 1 | 130 | 2.05 | 3.90 |
|  | Mexico | 18.21 | 1.47 | 22.25 | 0 | 124 | 2.11 | 4.09 |
|  | Peru | 34.21 | 2.09 | 31.52 | 0 | 191 | 1.97 | 5.42 |
|  | UK | 49.50 | 3.68 | 55.54 | 1 | 342 | 2.36 | 6.09 |
|  | Germany | 19.55 | 1.87 | 28.17 | 2 | 174 | 2.20 | 4.85 |
|  | Canada | 22.57 | 1.33 | 20.16 | 2 | 102 | 1.32 | 1.18 |
| Cumulative | United States | 16213.62 | 813.06 | 12276.95 | 2 | 29603 | -0.27 | -1.70 |
|  | Brazil | 5120.62 | 275.43 | 4158.97 | 2 | 10508 | -0.08 | -1.71 |
|  | Spain | 5025.65 | 189.29 | 2858.17 | 1 | 7496 | -0.76 | -1.15 |
|  | France | 2688.84 | 108.30 | 1635.36 | 2 | 4114 | -0.62 | -1.36 |
|  | Colombia | 1573.14 | 108.64 | 1640.43 | 1 | 4021 | 0.36 | -1.65 |
|  | Mexico | 1342.53 | 91.86 | 1387.13 | 2 | 3637 | 0.44 | -1.52 |
|  | Peru | 1682.86 | 95.24 | 1438.13 | 1 | 3643 | 0.03 | -1.69 |
|  | UK | 2704.95 | 86.01 | 1298.76 | 2 | 3730 | -0.97 | -0.68 |
|  | Germany | 2670.11 | 89.45 | 1350.60 | 2 | 3676 | -1.02 | -0.64 |
|  | Canada | 992.54 | 35.56 | 536.96 | 2 | 1460 | -0.74 | -1.13 |
